# Results of an Online Survey on Intensive Care Management of Patients with Aneurysmal Subarachnoid Hemorrhage in German-Speaking Countries

**DOI:** 10.3390/jcm13247614

**Published:** 2024-12-13

**Authors:** Anisa Myftiu, Lisa Mäder, Ilia Aroyo, Rainer Kollmar, on behalf of the IGNITE Study Group & DIVI Section Studies & Standards

**Affiliations:** 1 Department of Neurology and Neurintensive Care Medicine, Academic Hospital Darmstadt, 64283 Darmstadt, Germany; anisa.myftiu@mail.klinikum-darmstadt.de (A.M.); lisa.maeder@mail.klinikum-darmstadt.de (L.M.); ilia.aroyo@mail.klinikum-darmstadt.de (I.A.); 2 Department of Neurology, University Hospital Erlangen, Neurologische Universitätsklinik Erlangen, Friedrich-Alexander Universität Erlangen Nuremberg, 91054 Erlangen, Germany

**Keywords:** subarachnoid hemorrhage, intensive care medicine, multimodal monitoring, vasospasm

## Abstract

**Background:** The clinical course of patients with aneurysmal SAH (aSAH) is often dynamic and highly unpredictable. Since its management varies between hospitals despite guidelines, this survey aimed to assess the current state of intensive care treatment for aSAH in the German-speaking region and provide insights that could aid standardization of care for aSAH patients in the intensive care setting. **Methods:** From February 2023 to April 2023, medical professionals of the German Interdisciplinary Association for Intensive Care and Emergency Medicine (DIVI), the Initiative of German Neuro-Intensive Trial Engagement (IGNITE) network and manually recorded clinics with intensive care units were invited to participate in a standardized anonymous online questionnaire including 44 questions. The questionnaire was validated in multiple steps by experts of different specialties including those from the DIVI. A descriptive data analysis was carried out. **Results:** A total of 135 out of 220 participants answered the survey completely. The results showed that most patients were treated in anesthesia-led intensive care units at university and maximum care hospitals. Aneurysms were usually treated within 24 h after bleeding. If vasospasm was detected, induced hypertension was usually implemented as the first treatment option. In refractory vasospasm, interventional spasmolysis with calcium antagonists was usually carried out (81%), despite unclear evidence. There were significant discrepancies in blood pressure target values, particularly after aneurysm repair or after delayed cerebral ischemia (DCI), as well as in hemoglobin limit values for erythrocyte substitution. Despite the limited level of evidence, most institutions used temperature management (68%), including hypothermia (56%), for severe cases. **Conclusions:** While we anticipated variations between individual intensive care facilities, our survey identified numerous similarities in the treatment of aSAH patients. Methods such as interventional spasmolysis and temperature management were used frequently despite limited evidence. Our results can serve as a fundamental framework for formulating recommendations for intensive care treatment and planning of multicenter studies.

## 1. Introduction

The intensive care management of spontaneous aneurysmal subarachnoidal hemorrhage (aSAH) is complex and challenging due to the individual dynamics of the disease and limited applicable recommendations. Major challenges include the occurrence of delayed cerebral ischemia (DCI) and vasospasm. Up to 30% of patients with aSAH develop DCI within a time frame of 4 to 14 days after initial bleeding [1,2]. Vasospasm occurs angiographically in 70% of patients and is associated with DCI [3,4]. Up to 50% of patients with DCI suffer from ischemic stroke or death [2,4]. Therefore, prevention, detection, and treatment of vasospasm and DCI are important issues for intensive care management. However, the evidence for these measures is limited and therefore treatment of aSAH patients varies between different facilities [5,6]. These include the application of ultrasound, CT-techniques such as perfusion CT, blood pressure management, use of pharmacological agents for prevention and treatment of vasospasm as well as interventional techniques. Temperature management is another important and debated factor for intensive care after aSAH. Experimental data indicate multifactorial neuroprotective effects in focal cerebral ischemia and aSAH [7,8,9,10]. Moreover, first clinical investigations indicate beneficial effects in induced hypothermia for severe aSAH [11,12]. However, recent data on fever prevention in different kinds of stroke showed reduction of fever burden, but no improved outcome [13]. 

German S1 guidelines date back to 2012 and include only few recommendations for intensive care treatment for these patients [14]. Even recent international guidelines can only give recommendations of limited evidence for intensive care of aSAH of specific life-threatening scenarios [1]. 

Consequently, specialized or multidisciplinary intensive care units may possess markedly distinct objectives and therapeutic approaches when confronted with identical scenarios of hemodynamic monitoring, vasospasm, and delayed cerebral ischemia. As frequently reported, the outcome after aSAH is associated with annual treatment rates and therefore expertise. The wide implementation of interventional neuroradiologist due to the standard use of thrombectomy for embolic stroke might act as an accelerator for more aggressive treatment for vasospasm and DCI [15]. Moreover, there seems to be a continuous interest in the management of body core temperature. While first clinical investigations indicated beneficial effects of therapeutic hypothermia, a recently published trial could not show improved outcome when fever was avoided in acute neurovascular injury [16]. Since most aSAH patients are treated in high volume centers, it would be advantageous to establish a standardized treatment approach based on their experience. Our objective was to evaluate the current treatment status of patients with aneurysmal subarachnoid hemorrhage in German-speaking countries. Moreover, we aimed and to identify comparable routine targets and treatment approaches based on daily routine and practical ICU management as well as to identify significant disparities. These data could be used as a guiding framework for general intensive care treatment recommendations, which are currently formulated within the German Interdisciplinary Association for Intensive Care and Emergency Medicine (DIVI) neurological section and the Initiative of German Neuro-Intensive Trial Engagement (IGNITE) network which is part of the German Society of Neurointensive Care (DGNI) and performs mono- and multicenter clinical trials. Information of this survey can help to start a forthcoming registry, and multicenter studies initiated by the DIVI and IGNITE study groups.

## 2. Methods

In collaboration with an interdisciplinary team composed of neurologists, neurosurgeons, neuroradiologists, neurointensivists and anesthesiologists from various hospitals in Germany, Switzerland, and Austria, we (as part of the DIVI Group Studies & Standards) developed an online questionnaire comprising 13 mandatory questions (a total of 44 questions, including closed and partially open questions, and some with multiple choices). These were intended to document the intensive care management of patients with SAH in various hospitals in German-speaking regions. The covered subjects included structural conditions, aneurysm care, blood pressure targets, cerebrospinal fluid (CSF) drainage management, airway management and sedation, general measures, surveillance, and vasospasm treatment. The intensive care physicians of the Darmstadt Hospital and the DIVI SAH study group assessed and validated the draft after an internal audit and made necessary adjustments. As the survey was conducted anonymously and did not involve the collection of any patient data, ethical approval was not required. A link to the anonymized online questionnaire created via the website Netigate.se was sent via email internally to the DIVI and IGNITE mailing lists as well as manually recorded clinics with neurointensive care units in Germany, Switzerland and Austria. Answering the survey took an average of 13 min. The results were collected on the website Netigate.se and then descriptively analyzed by Netigate. The questionnaire was open for answers from 1 February 2023 to 30 April 2023. These questions have been added to the Supplementary Material S1 of this manuscript.

## 3. Results

### 3.1. Profile of Respondents and Participating Hospitals

**Figure 1:**
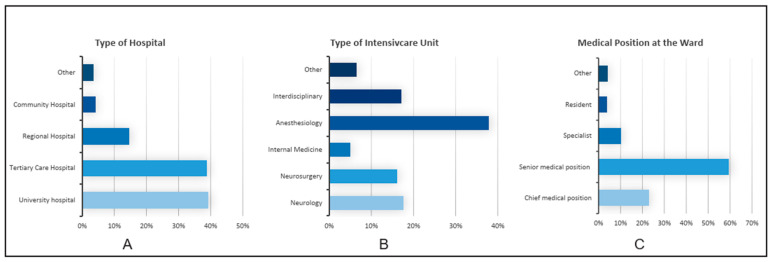
Results of the questions regarding the type of hospital, ICU, and medical position on the ward. The X-axis represents the percentage of answers we received for each answer aligned on the Y-axis. (**A**) indicates the proportions of different types of hospitals. (**B**) shows what medical discipline leads the ICU. (**C**) highlights the contribution of the medical position of the respondents.

The survey was fully completed by a total of 135 out of 220 participants. A significant proportion of the participants, 59%, held senior medical positions, and 23% held chief medical positions. Approximately 78% of them worked in university clinics and tertiary care hospitals. From these, 43% treated over 50 SAH cases per year, and 25% treated over 20 SAH cases per year. Patients with SAH were most frequently treated in an anesthesiological intensive care unit (ICU) (Figure 1A–C). According to the survey, 79% of the respondents reported daily visits by anesthesiologists, neurologists, neurosurgeons, and microbiologists. In addition, 35% of respondents reported that intensive care decisions were made by interdisciplinary teams, while 30% reported that decisions were made solely by neurosurgeons.

### 3.2. Aneurysm Treatment

Angiography, endovascular therapy, and surgical treatment: According to the survey, 76% of the respondents reported that the aneurysm was treated within 24 h of the patient’s presentation, 21% within 24 to 48 h, and only 3% over 48 h. Sixty-one percent of those surveyed could perform angiography 24 h a day, 7 days a week, and only 25% performed angiography only during regular working hours. Endovascular care was accessible 24 h a day for 53% of those surveyed, whereas 29% of respondents had access only during their regular working hours. Aneurysms could be treated with clipping continuously in 60% of cases, and in 27% of cases, only during regular working hours.

High care and intermediate care treatment: Within the initial 14 days, 31% of patients were exclusively treated in the intensive care unit, 51% were transferred to the stroke unit or intermediate care unit for mildly affected patients, and 19% were transferred to a normal ward for patients who were clinically stable.

### 3.3. Blood Pressure Management

Blood pressure was monitored via arterial lines in 86% of those surveyed. Extended hemodynamic monitoring was carried out using a PiCCO catheter in 53% and regular point-of-care (POCUS) ultrasound examinations (echocardiography and vena cava ultrasonography) were performed in 31%.

**Figure 2:**
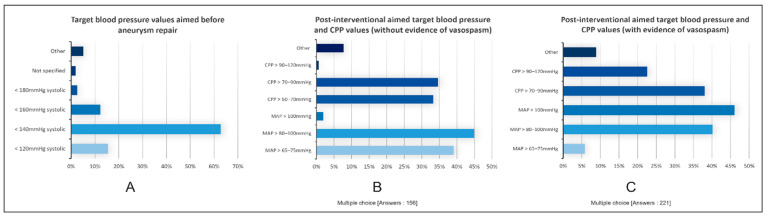
Results of the questions regarding target blood pressure and CPP values in patients with aSAH. The X-axis represents the percentage of responses we received for each answer shown on the Y-axis (systolic blood pressure, CPP and MAP). (**A**) indicates the target systolic blood pressure before treatment of the aneurysm. (**B**) indicates target blood pressure after aneurysm repair when no signs of vasospasm appeared. (**C**) shows target blood pressure after aneurysm repair under the condition of vasospasm. For questions B and C, multiple-choice answers were allowed. Most respondents indicated they would aim to keep the blood pressure before aneurysm repair below 140 mmHg, post-intervention MAP > 90–100 mmHg, and post-intervention with vasospasm MAP > 100 mmHg and CPP > 70–90 mmHg.

Target values: The target blood pressure value before aneurysm treatment was <140 mmHg systolic in 63% of the clinics, and the other values were <120 mmHg (15%) or <160 mmHg (12%). In the postinterventional phase without evidence of vasospasm, 45% of respondents aimed for a mean arterial pressure (MAP) of >80 to 100 mmHg, and 39% aimed for a MAP of >65 to 75 mmHg. The cerebral perfusion pressure (CPP) strived for in 35% of the clinics was >70 to 90 mmHg, and in 33% of the clinics, the CPP was >50 to 70 mmHg. If postinterventional vasospasm developed, a MAP adjustment to >100 mmHg was targeted in 46% and between 80 and 100 mmHg in 40% (Figure 2A–C). 

### 3.4. Airway Management and Sedation

Not severely affected patients: 99% of the participants indicated that prompt postinterventional extubation was planned for patients who were not severely affected (World Federation of Neurological Surgeons Scale/WFNS I–III) to facilitate a clinical assessment of the patient. The target Richmond Agitation-Sedation Scale (RASS) score for each patient was determined individually in 33% of cases, the target RASS score was −1/−2 for 32% of cases. 

Severely affected patients: According to the survey, 61% of patients who were severely affected (WFNS IV–V) were also promptly extubated after the intervention. Thirty-nine percent of the individuals surveyed opted to manage the sedation depth individually, with 20% aiming for deeper sedation (RASS score of −3/−4). In the initial treatment for sedation, respondents used primarily sufentanil/remifentanil and propofol, followed by benzodiazepines and esketamine.

### 3.5. External Ventricular Drainage (EVD) Management and ICP Probing

**Figure 3:**
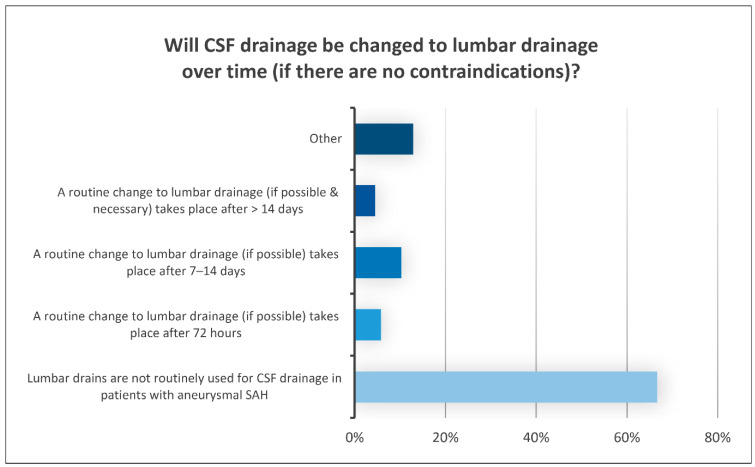
Results: Results of the question regarding the transition from CSF drainage via EVD to lumbar drainage over time. The X-axis represents the percentage of responses we received for each answer shown on the Y-axis (different time frames for the change in EVD usage), indicating that lumbar drains are still not part of routine practice.

EVD management included continuous CSF drainage via the water column in 76% of the hospitals. Discontinuous drainage was used in the remaining clinics using the ICP as a goal parameter or a fixed drainage volume amount per hour. The process of weaning from the EVD was conducted based on neurosurgical criteria (48%) or on the delivery volume (35%). Switching to lumbar drainage (LD) (67%) was not routine practice, as depicted in Figure 3. In 38% of hospitals, the examination for an infection of the CSF was solely conducted in cases of clinical suspicion. In 36% of the clinics, it was routinely conducted twice a week.

### 3.6. Epileptic Seizures and EEG Monitoring

Fifty-eight percent of participants could perform continuous EEG monitoring in their intensive care units, and 40% were able to perform discontinuous EEG monitoring. EEG monitoring was performed in 39% of patients with an epileptic seizure; routine EEG monitoring was not applied in 42% of patients. Prophylactic anticonvulsive medication was not part of routine treatment in 99% of the cases. 

### 3.7. Vasospasm

Vasospasm prophylaxis and detection: Systemic nimodipine was started in two-thirds of the respondents within 24 h after SAH. Nimodipine was typically administered orally (77%), through the gastric tube (69%) or intravenously (54%). Vasospasm was most frequently investigated by regular transcranial ultrasound (TCD) (90%) and clinical examination (71%), followed by CT angiography (60%) and CT perfusion (60%). Other procedures, such as brain tissue oxygen monitoring (PtiO2) (25%) or microdialysis (8%), were rarely used. TCD was performed once per day in 61% of patients and several times per day in 14% or several times per week in 14%.

**Figure 4:**
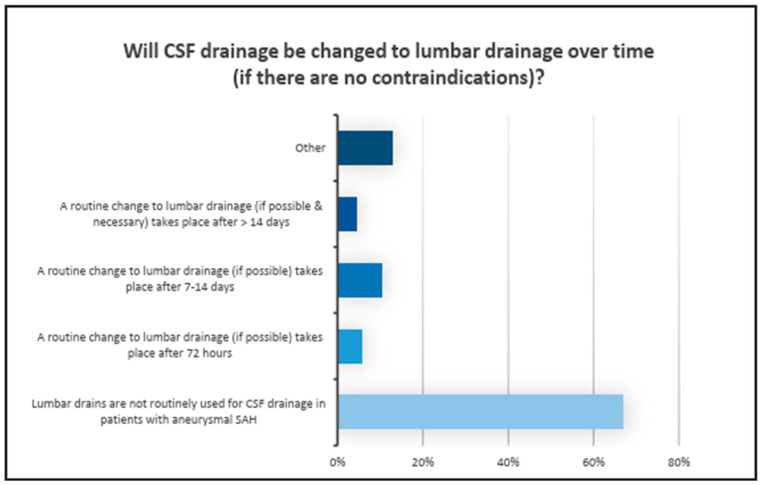
Results of the question regarding treatment options after vasospasm detection. The X-axis represents the percentage of responses we received for each answer shown on the Y-axis, which included various treatment options. Induced hypertension was the most common treatment, followed by interventional spasmolysis with calcium antagonists.

Treatment of detected vasospasm: The following therapies were considered for the treatment of vasospasms: induced hypertension (84%), interventional spasmolysis with calcium antagonists (81%) or neuroradiological percutaneous transluminal angioplasty (PTA) (53%) (Figure 4). The criteria for interventional therapy to address vasospasm included evidence of vasospasm and the inability to respond to conservative measures in 56% of those surveyed. Fifty-one percent of respondents waited for clinical evidence of delayed cerebral ischemia, as shown by a new focal neurological deficit or decrease in consciousness. Overall, 45% of respondents indicated that imaging evidence of relevant vasospasm was the index parameter for interventional therapy.

Recurrent or persistent vasospasm: In the event of recurrent or persistent vasospasm, induced hypertension was used as a first-line therapy in 75% of the participants. Another interventional spasmolysis procedure using calcium antagonists (60%) was performed as the second-most prevalent option. Twenty-five percent of those surveyed chose percutaneous balloon dilatation for refractory vasospasm. Interventional spasmolysis with calcium antagonists and induced hypertension were also employed as second-line therapies in 53% and 38% of cases, respectively, when first-line therapy proved unsuccessful.

### 3.8. General Measures

Prophylaxis for venous thrombosis: According to the survey, 76% of the respondents stated that prophylactic measures for venous thrombosis were initiated after the intervention (clipping/endovascular treatment) within a period of 24 to 48 h.

**Figure 5:**
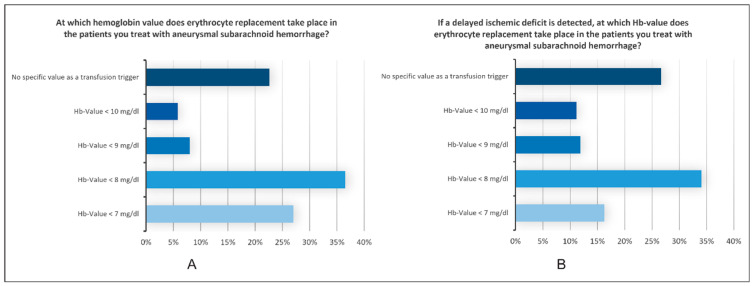
Results of the question regarding hemoglobin values and erythrocyte replacement in patients with aSAH, either without (**A**) or after (**B**) the detection of delayed ischemic deficit. The X-axis represents the percentage of responses we received for each answer shown on the Y-axis, which included different hemoglobin value thresholds. Most respondents indicated they would initiate erythrocyte replacement at hemoglobin levels below 8 mg/dL, both with and without DCI.

Hb Management: In patients with aneurysmal SAH, the indication for erythrocyte transfusion was set by 36.5% of the participants, with an Hb value of 8 mg/dL in 27% as 7 mg/dL, and no specific trigger for 23% of the participants. In the case of delayed ischemic deficits, 34% of those surveyed substituted for erythrocytes when Hb fell below <8 mg/dL. In 27% of those surveyed, there was no specific Hb target value, even in this case (Figure 5).

Temperature management: Body temperature was measured mainly intravesically (80%) and continuously (67%). Moreover, 41% of the surveyed physicians indicated that temperature-lowering measures were initiated when body temperature was ≥38 °C, and 27% of the physicians indicated that temperature-lowering measures were initiated when body temperature reached ≥37.5 °C. Antipyretic measures included pharmacological fever reduction (93%), cooling blankets/cooling pads (76%), washing with calf wraps or peppermint (71%), mechanical techniques (feedback-controlled devices such as adhesive pads and catheter cooling) (64%), and cold infusions (55%). Prophylactic controlled normothermia was employed by 56% of the individuals surveyed.

Use of antiplatelet therapy: Prophylactic administration of platelet function inhibitors after DCI occurred was usually not given (74%).

Mobilization: In the absence of contraindications, early mobilization occurred within 24 h after aneurysm treatment in 35%, within the first 24–72 h in 32%, and more than 72 h after the event in 21% of patients.

Diet: In two-thirds of participants, nutrition started with a predominantly normocaloric target (89%).

## 4. Discussion 

Our survey provides valuable insights into the current practices of intensive care treatment for aneurysmatic subarachnoid hemorrhage in German-speaking countries. It offers a comprehensive overview of hospital characteristics, including the admission and treatment of aSAH patients, the management of complications, and specific therapeutic strategies associated with intensive care management. Of importance, we report a high surprisingly high rate for the use of interventional techniques for vasospasm and DCI. Moreover, the majority of respondents use targeted temperature management and sometimes induced hypothermia in severely affected aSAH patients. Both treatment options are not recommended as level Ia or Ib evidence in the current guidelines [1].

### 4.1. Profile of Respondents and Participating Hospitals

Most of our survey findings align closely with the current German and American Heart Association/American Stroke Association (AHA/ASA) recommendations for treating severe aSAH patients in high-level, tertiary care hospitals or university settings [1,17]. Patients experiencing severe aSAH are predominantly treated in high-level care hospitals or university settings, allowing for aneurysm treatment typically within 24 h. These findings align with the 2017 recommendations from the German Society for Neurology (DGN), which emphasize the importance of acute aSAH treatment in accredited centers with proficient vascular neurosurgeons and interventional neuroradiologists [14]. Our results are concordant to data of a recently published survey in which endovascular neurosurgeons, neuroradiologists and neurologists were contacted by the Society of NeuroInterventional Surgery (SNIS) and the European Society of Minimally Invasive Neurological Therapy (ESMINT) [18]. 

### 4.2. Aneurysm Treatment and Allocation of the Patients

Treatment of ruptured aneurysm performed up to day 1 was in 76% in our study compared to 71% as reported by Guenego [18]. Both surveys support that the aneurysm is secured within 24 h in daily practice. This is in accordance to the current guidelines of the AHA/ASA which recommend prompt treatment of aneurysms, ideally within 24 h, in order to improve outcome [1], while a broader timeframe of up to 72 h is provided by the German S1 guideline [14]. The potential benefit of early endovascular therapy within 24 h for high-grade aSAHs (Hunt-Hess IV–V/WFNS IV–V) has recently been indicated by a retrospective cohort study from China improving patient prognosis [19]. 

Most patients are admitted to the intensive care unit or intermediate care unit during the critical phase. Of the participating centers, 68% treated over 25 patients with aSAH per year (43% treated over 50 aSAH, 25% treated over 20 aSAH cases per year). These data are in accordance with the survey of Guenego in which 71% of the centers treated more than 25 aSAH patients per year. Interestingly, the majority of patients treated in German speaking countries are treated in an anesthesiologic intensive care unit. Neurological expertise is still incorporated in German ICUs by daily visits by anesthesiologists, neurologists, neurosurgeons, and microbiologist. However, only 35% of respondents reported that intensive care decisions were made by interdisciplinary teams. Since major clinical problems such as the detection and management of vasospasm and DCI as well as CSF management require a multidisciplinary approach, we consider written SOPs in which this team effort is addressed. 

### 4.3. Blood Pressure Management

For the management of blood pressure, we observed a huge variation in target values which might display guidelines.

Methods for monitoring blood pressure were also different between the centers. Usually, arterial pressure curve measurements is more prevalent than pulse contour cardiac output (PiCCO) monitoring. However, clinical data indicated that goal directed hemodynamic therapy (GDHT) including PiCCO techniques improves outcome in high grade SAH [20]. In our survey, extended hemodynamic monitoring was carried out using a PiCCO catheter in 53%, which is a particularly high rate. Interestingly, the referred survey for treatment of vasospasm did not include specific questions about hemodynamic monitoring and treatment [18].

Despite limited evidence, the majority of the hospitals surveyed utilized a target value of 140 mmHg systolic prior to aneurysm treatment and post-interventional MAP values of >65 mmHg or, in the event of vasospasm detection, >80 mmHg. Given that the CPP value can influence actual cerebral blood flow, it seems prudent to implement a CPP-controlled therapy. The AHA/ASA guidelines emphasize the importance of avoidance of blood pressure fluctuation and recommend a gradual reduction of the blood pressure in case of values above 180–200 mm Hg before aneurysm obliteration. They also recommend a strict avoidance of hypotension [1]. 

### 4.4. Detection and Management of Vasospasm and DCI

In our survey, vasospasm was most frequently detected by regular transcranial ultrasound (TCD) (90%) and clinical examination (71%), followed by CT angiography (60%) and CT perfusion (60%). However, TCD was performed daily only by 61% of responders. Vasospasm detection is commonly conducted using regular ultrasound assessments and clinical evaluations, although the specific protocols and frequency of these assessments can vary.

Prevention and treatment of vasospasm by systemic application of nimodipine was started in two-thirds of the respondents within 24 h after SAH. Nimodipine was typically administered orally (77%). These results are in accordance to other data [18]. As expected, induced hypertension was the earliest and most frequent measure when vasospasm and/or DCI was suspected or approved. As an escalation therapy, 81% of responders used interventional spasmolysis with calcium antagonists or neuroradiological percutaneous transluminal angioplasty (PTA) (53%). Compared to a German survey from 2006, this rate increased from 30% [21]. This frequent use of interventional treatment might be explained by the high expertise in large neurovascular centers, routine implementation of thrombectomy for acute large vessel occlusions in ischemic stroke, and a need for treatments more than hemodynamic measures and systemic nimodipine. Our results are in accordance to those of Guenego who reported endovascular treatment in 89% when deficits were associated with proven vasospasm/DCI. In this study, mechanical angioplasty was considered the most effective endovascular treatment by 65% of neurointerventionalists, while our survey also reported 53% of responders to use PTA. 

Prevention and treatment of occlusive or/and malresportive hydrocephalus is another key point for intensive care treatment. While the initial management for occlusive hydrocephalus certainly includes external ventricular drainage, further management is an ongoing debate on the intensive care unit. There are no clear recommendations for management and weaning from the EVD. There is also a dearth of evidence regarding the optimal drainage strategy, whether it be continuous or discontinuous. A multi-institutional American survey found that for treated aneurysms, continuous drainage is usually performed in conjunction with a gradual weaning strategy [22]. Rapid EVD weaning resulted in shorter lengths of stay, but did not provide clinical benefits compared to gradual weaning [23]. Another point was the routine implementation of lumbar drainage (LD). Despite the Early DRAIN Study indicating that prophylactic placement of LD within 72 h of SAH reduces unfavorable outcomes at six months, its usage varied widely [24]. 

Invasive multi-modal monitoring (PtiO2, microdialysis), aimed at providing real-time information to detect delayed cerebral ischemia events before irreversible damage occurs, was rarely used in practice. This is likely due to insufficient evidence on its impact on long-term clinical outcomes in high-grade aSAH patients [25]. 

Prophylactic controlled normothermia was commonly employed by more than half of the surveyed physicians, indicating a preference for maintaining stable body temperatures within normal ranges. This result is surprising, given the limited data on targeted temperature management (TTM) after aSAH and its frequent side effects. Most studies on TTM have small patient numbers, lack control groups, lack randomization, and have varied treatment protocols. Side effects include shivering requiring pharmacological intervention, prolonged sedation, increased duration of mechanical ventilation, and extended ICU stay [1]. Despite these limitations, a significant proportion of intensivists still consider temperature to be a major treatment target and use TTM. 

Certainly, our study has several limitations especially due to the characters of an online survey. Most of all, respondents may be biased by the selection and phrasing of the questionnaire, and correctness of the answers cannot be proofed. Another limiting factor in online surveys is the population to which they are distributed cannot be described. However, in our study we cover a large proportion of hospitals and received answers mostly from chief or senior physicians.

To summarize, we presented a comprehensive examination of current intensive care practices for aSAH treatment in Germany, Austria, and Switzerland. Our findings demonstrate the alignment of aSAH therapy with established guidelines, highlighting the importance of specialized care in accredited centers for acute aSAH patients. The discrepancies revealed in our survey emphasize the need for further research and standardization to improve patient outcomes. Establishing a registry to compare treatment algorithms and targets presents a potential avenue for future research, contributing to the collective effort to advance evidence-based practices in aSAH care.

## 5. Conclusions

Our survey provides insight into the real-world, practical intensive care management of patients suffering from aSAH in German-speaking countries. Some intensive care management parameters differed within the survey. Surprisingly, despite limited evidence, interventional treatments for vasospasm and DCI are widely used. This is true even in the context of temperature management, as numerous hospitals employ prophylactically controlled normothermia or extend treatment to hypothermia. Our data can be used to debate and adjust standards for aSAH care within institutions. As a next step, we will create practical recommendations within the DIVI aSAH study group and IGNITE to guide major aspects of aSAH care. As we observe a particular will to address treatment options with high impact, such as interventional therapy or TTM, creating a register of the various treatment concepts and target values could contribute to more evidence.

## Data Availability

The datasets used and/or analyzed during the current study are available from the corresponding author upon reasonable request.

## Supplementary Materials

The following supporting information can be downloaded at: https://www.mdpi.com/article/10.3390/jcm13247614/s1, Supplementary Material S1: Questions of the online survey.

## Abbreviations

AHA/ASA	American Heart Association/American Stroke Association
aSAH	aneurysmal SAH
CPP	cerebral perfusion pressure
CSF	cerebrospinal fluid
DCI	delayed cerebral ischemia
DIVI	German interdisciplinary association for intensive care and emergency medicine
DGN	German Society for Neurology
EVD	external ventricular drainage
ESMINT	European Society of Minimally Invasive Neurological Therapy
IGNITE	the Initiative of German NeuroIntensive Trial Engagement
MAP	mean arterial pressure
LD	lumbar drainage
PTA	percutaneous transluminal angioplasty
RASS	Richmond Agitation-Sedation Scale
TCD	transcranial ultrasound
WFNS	World Federation of Neurological Surgeons Scale
