# Supplementary material for: Results of an Online Survey on Intensive Care Management of Patients with Aneurysmal Subarachnoid Hemorrhage in German-Speaking Countries
Source: J Clin Med. 2024 Dec 13;13(24):7614. doi: 10.3390/jcm13247614 (PMC11676747; doi:10.3390/jcm13247614)
Supplement: Supplementary file 1 [file jcm-13-07614-s001.zip › jcm-3214028-supplementary.pdf]

S1: Supplementary data: Questions of the online survey

1. General information **In which country do you work?**

- Germany
- Austria
- Switzerland

2. General information **Please assign yourself to a federal state:**

- Baden-Württemberg
- Bavaria
- Berlin
- Brandenburg
- Bremen
- Hamburg
- Hesse
- Mecklenburg-Western Pomerania
- Lower Saxony
- North Rhine-Westphalia
- Rhineland-Palatinate
- Saarland
- Saxony-Anhalt
- Saxony
- Schleswig-Holstein
- Thuringia

3. General information **Please assign yourself to a federal state:**

- Burgenland
- Carinthia
- Lower Austria
- Upper Austria
- Salzburg
- Styria
- Tyrol
- Vorarlberg

4. General information **Please assign yourself to a canton:**

- Aargau
- Appenzell Ausserrhoden
- Appenzell Innerrhoden
- Basel-Land
- Basel-Stadt
- Bern
- Freiburg
- Geneva
- Glarus
- Graubünden
- Jura
- Lucerne
- Nidwalden

- Neuenburg
- Obwalden
- St. Gallen
- Schaffhausen
- Schwyz
- Solothurn
- Ticino
- Thurgau
- Uri
- Vaud
- Valais
- Train
- Zurich

5. General information **What type of hospital do you work at?**

- basic care
- standard care
- maximum care
- university hospital
- Other

5. General information **Other**

Answer

5. General information **Which department does the intensive care unit you work in belong to?**

- neurology
- neurosurgery
- Internal Medicine
- anesthesia
- Interdisciplinary
- Other

5. General information **Other**

Answer

5. General information **What is your role?**

- chief physician
- senior physician
- specialist doctor
- assistant doctor
- Other

5. General information **Other**

Answer

5. General information **How often do interdisciplinary visits take place?**

- Daily
- Several times a week
- Once a week
- Never

- Other

5. General information **Other**

Answer

5. General information **Which departments take part in the interdisciplinary visits? (Multiple answers possible)**

- neurology
- neurosurgery
- microbiology
- cardiology
- nephrology
- Other

5. General information **Other**

Answer

5. General information **Who makes the intensive care decisions regarding aneurysmal SAH patients on your ward?**

- anesthesia
- internal Medicine
- interdisciplinary
- neurosurgery
- neurology
- neuroradiology/ Radiology
- Other

5. General information **Other**

Answer

6. Treatment of aneurysmal subarachnoid hemorrhages **How many subarachnoid hemorrhages are treated on average in your ICU each year?**

- < 5 cases per year
- 5 – 20 cases per year
- > 20 cases per year
- >50 cases per year

6. Treatment of aneurysmal subarachnoid hemorrhages **When does an aneurysm treatment usually take place in your hospital after the patient has presented it?**

- <24h
- 24-48 hours
- 48-72 hours
- > 72 hours

6. Treatment of aneurysmal subarachnoid hemorrhages

Until what time do you care for a stable subarachnoid hemorrhage?
A with an angiography

- 24/7
- During regular working hours
- Not applicable

B With an endovascular treatment

- 24/7
- During regular working hours
- Not applicable

C With a clipping

- 24/7
- During regular working hours
- Not applicable

6. Treatment of aneurysmal subarachnoid hemorrhages

If you selected "Not applicable", please explain here:

Answer

6. Treatment of aneurysmal subarachnoid hemorrhages

If there is no interventional option for aneurysm treatment on the day of admission - for aneurysms with an increased risk of bleeding and no other contraindications - is short-term (<72h) therapy with tranexamic acid or aminocaproic acid performed?

- Yes, always
- Yes, often
- No

6. Treatment of aneurysmal subarachnoid hemorrhages

Are all patients with aneurysmal subarachnoid hemorrhage treated in the intensive care unit for at least 14 days?

- Yes, in intensive care.
- No, transfer of mildly affected patients to SU/IMC is possible.
- No, if clinically stable, treatment on a normal ward is also possible.

6. Treatment of aneurysmal subarachnoid hemorrhages

Do you have different intensive care treatment algorithms?
based on the clinical condition of the patients? (Multiple answers possible)

- Yes, according to WFNS or Hunt & Hess grade at admission
- Yes, depending on age and previous illnesses
- No

7. Treatment of aneurysmal subarachnoid hemorrhages

What target blood pressure values are aimed for
in patients with SAH before aneurysm repair?

- < 120mmHg systolic
- < 140mmHg systolic
- < 160mmHg systolic
- < 180mmHg systolic
- Unspecified
- Other

7. Treatment of aneurysmal subarachnoid hemorrhages

Other

Answer

7. Treatment of aneurysmal subarachnoid hemorrhages

What target blood pressure and CPP (cerebral perfusion pressure) values are aimed for in patients with SAH postinterventionally (without evidence of vasospasm)? (Multiple answers possible)

- Mean arterial pressure > 65 - 75mmHg
- Mean arterial pressure > 80 - 100mmHg
- Mean arterial pressure > 100mmHg
- CPP > 50-70mmHg
- CPP > 70 - 90mmHg
- CPP > 90-120mmHg
- Other

7. Treatment of aneurysmal

Other

subarachnoid hemorrhages

Answer

7. Treatment of aneurysmal subarachnoid hemorrhages

Which statement is correct regarding airway management of patients with aneurysmal subarachnoid hemorrhage?

A For patients with WFNS I – III

- A timely post-interventional extubation is aimed for in the
- absence of contraindications (e.g. intracranial pressure) in order
- to enable a clinical assessment of the patient.
- Initially, prolonged ventilation is preferred due to the risk of
- vasospasm and possible necessary interventions.

B For patients with WFNS IV – V

- A timely post-interventional extubation is aimed for in the absence of contraindications (e.g. intracranial pressure) in order to enable a clinical assessment of the patient.
- Initially, prolonged ventilation is preferred due to the risk of vasospasm and possible necessary interventions.

7. Treatment of aneurysmal subarachnoid hemorrhages

What is the target RASS for patients with sedation?

A Patients with WFNS I – III

- RASS 0
- RASS -1/ -2
- RASS -3/-4
- RASS -5
- Individual decision

B Patients with WFNS IV – V

- RASS 0
- RASS -1/ -2
- RASS -3/-4
- RASS -5
- Individual decision

7. Treatment of aneurysmal

Which medications are used for sedation in patients with aneurysmal subarachnoid hemorrhage on your first-line ward? (Multiple answers possible)

subarachnoid hemorrhages

- Propofol
- benzodiazepines
- esketamine
- Sulfentanil/Fentanyl/Remifentanyl
- isoflurane/sevoflurane
- α2 antagonists
- Other

7. Treatment of aneurysmal subarachnoid hemorrhages

Other

Answer

8. Treatment of aneurysmal subarachnoid hemorrhages

When is thrombosis prophylaxis routinely started on your ward in patients with aneurysmal subarachnoid hemorrhage?

- Immediately after admission ≤ 24h
- After intervention- clipping/ endovascular treatment - (24 – 48h)
- > 48h
- Other

8. Treatment of aneurysmal subarachnoid hemorrhages

Other

Answer

8. Treatment of aneurysmal subarachnoid hemorrhages

In addition to the placement of an EVD (if indicated), is an ICP probe also placed?

- No
- Yes, routinely
- Yes, in analgosedated patients
- Yes, in special cases

8. Treatment of aneurysmal

Yes, in special cases

subarachnoid hemorrhages

Answer

8. Treatment of aneurysmal subarachnoid hemorrhages

Is a CT scan performed immediately after the EVD has been placed?

- No
- Yes, always
- Depending on the case

8. Treatment of aneurysmal subarachnoid hemorrhages

How is CSF drainage performed via the inserted EVD?

- Continuously above water column
- Discontinuous with millimeter quantity/hour
- Apparatus (e.g. Liquorguard®)
- No fixed regime
- Other

8. Treatment of aneurysmal subarachnoid hemorrhages

Other

Answer

8. Treatment of aneurysmal subarachnoid hemorrhages

When does weaning from the EVD usually begin after it has been installed?

- According to flow rate
- According to neurosurgical standards
- Time limit: < 7 days
- Time limit: < 14 days
- Other

8. Treatment of aneurysmal subarachnoid hemorrhages

Other

Answer

8. Treatment of aneurysmal subarachnoid hemorrhages

Will the CSF drainage be changed to a lumbar drainage over time (if there are no contraindications)?

- Lumbar drains are not routinely used for CSF drainage in patients with aneurysmal SAH.
- A routine change to a lumbar drain (if possible) takes place after 72 hours.
- A routine change to a lumbar drain (if possible) occurs after 7-14 days.
- A routine change to a lumbar drain (if possible & necessary) takes place after > 14 days.
- Other

8. Treatment of aneurysmal subarachnoid hemorrhages

Other

Answer

9. Treatment of aneurysmal subarachnoid hemorrhages

How often is the CSF tested for infections when a CSF drain is in place?

- Daily
- once a week
- 2 times a week
- No fixed examination scheme, indication based on clinical suspicion.
- Other

9. Treatment of aneurysmal subarachnoid hemorrhages

Other

Answer

9. Treatment of aneurysmal subarachnoid hemorrhages

Is it possible to record an EEG in patients with aneurysmal SAH in the intensive care ur

- Yes, continuously
- Yes, discontinuous
- No

9. Treatment of aneurysmal subarachnoid hemorrhages

Is EEG monitoring routinely performed in patients with aneurysmal SAH?

- o Yes, in all patients with aneurysmal SAH
- o Yes, in case of epileptic seizures
- o Yes, in analgosedated patients
- o No
- o Other

9. Treatment of aneurysmal subarachnoid hemorrhages

Other

Answer

9. Treatment of aneurysmal subarachnoid hemorrhages

Is anticonvulsant prophylaxis routinely performed in all patients with aneurysmal SAH

- o No
- o Yes, with the following anticonvulsant

9. Treatment of aneurysmal subarachnoid hemorrhages

Yes, with the following anticonvulsant

Answer

9. Treatment of aneurysmal subarachnoid hemorrhages

When is vasospasm prophylaxis with nimodipine started in patients with aneurysmal subarachnoid hemorrhage?

- o <24h
- o post-interventional
- o If there is evidence of vasospasm in transcranial ultrasound
- o If there is evidence of vasospasm in MR angiography or CT angiography

9. Treatment of aneurysmal subarachnoid hemorrhages

How is nimodipine administered prophylactically? (Multiple answers possible)

- Per os
- Via the gastric tube
- Intravenous
- Intraarterial
- Intrathecal

10. Treatment of aneurysmal subarachnoid hemorrhages

Is regular transcranial ultrasound diagnostics performed to obtain indications of possible cerebral vasospasms or to monitor their progression?

- No routine transcranial ultrasound diagnostics are performed.
- Transcranial ultrasound diagnostics are performed once a day.
- Transcranial ultrasound diagnostics are performed several times a day (e.g. once per shift).
- Transcranial ultrasound diagnostics are performed several times a week.
- Other

10. Treatment of aneurysmal subarachnoid hemorrhages

Other

Answer

10. Treatment of aneurysmal subarachnoid hemorrhages

How is vasospasm detection performed? (multiple answers possible)

- No routine vasospasm detection is performed.
- Clinical examination
- Regular transcranial duplex sonographic checks
- CT angiography
- CT perfusion
- MR/MRA perfusion
- ptiO2 probe
- microdialysis
- Other

10. Treatment of aneurysmal subarachnoid hemorrhages

Other

Answer

10. Treatment of aneurysmal subarachnoid hemorrhages

If you have vasospasm detection performed, how often is this done?

- Several times a day
- Daily
- above ground
- Other

10. Treatment of aneurysmal subarachnoid hemorrhages

Other

Answer

10. Treatment of aneurysmal subarachnoid hemorrhages

What therapy is carried out if vasospasm is detected? (multiple answers possible)

- Induced hypertension
- Induced hypervolemia
- Interventional spasmolysis with calcium antagonists
- Neuroradiological PTA
- Therapeutic hypothermia
- None, as there is no routine screening
- Other

10. Treatment of aneurysmal subarachnoid hemorrhages

Other

Answer

10. Treatment of aneurysmal subarachnoid hemorrhages

When is the indication for interventional vasospasm therapy made? (Multiple answers possible)

- For every detection of vasospasm
- If vasospasm is detected despite conservative measures (e.g. induced hypertension)
- If vasospasm is proven by imaging.
- After clinical evidence of delayed cerebral ischemia with of a new focal neurological deficit.
- No interventional vasospasm therapy is performed.

10. Treatment of aneurysmal subarachnoid hemorrhages

Which endovascular intervention is used in case of vasospasm?

- Interventional spasmolysis with calcium antagonists
- Interventional percutaneous transluminal angioplasty
- PTA with balloon system-noncompliant
- PTA with balloon system compliant
- Stentretriever/Comanechi
- No interventional vasospasm therapy is performed.

11. Treatment of aneurysmal subarachnoid hemorrhages

What is the first-line treatment if vasospasm recurs after previous treatment? (including interventional therapy, multiple answers possible)

- Induced hypertension
- Induced hypervolemia
- Interventional spasmolysis with calcium antagonists
- interventional percutaneous transluminal angioplasty
- PTA with balloon system-noncompliant
- PTA with balloon system compliant
- Stentretriever/ComanechiÒ
- Therapeutic hypothermia
- No further therapy
- Other

11. Treatment of aneurysmal subarachnoid hemorrhages

Other

Answer

11. Treatment of aneurysmal subarachnoid hemorrhages

What therapy is used as second-line if vasospasm recurs after previous therapy? (including interventional therapy, multiple answers possible)

- Induced hypertension
- Induced hypervolemia
- Interventional spasmolysis with calcium antagonists
- interventional percutaneous transluminal angioplasty
- PTA with balloon system-noncompliant
- PTA with balloon system compliant

- Stentretriever/ComanechiÒ
- Therapeutic hypothermia
- No further therapy
- Other

11. Treatment of aneurysmal subarachnoid hemorrhages

Other

Answer

11. Treatment of aneurysmal subarachnoid hemorrhages

What target blood pressure and CPP (cerebral perfusion pressure) values are aimed for in patients with SAH postinterventionally (with evidence of vasospasm)? (Multiple answers possible)

- Mean arterial pressure > 65 - 75mmHg
- Mean arterial pressure > 80 - 100mmHg
- Mean arterial pressure > 100mmHg
- CPP > 70 - 90mmHg
- CPP > 90-120mmHg
- Other

11. Treatment of aneurysmal subarachnoid hemorrhages

Other

Answer

11. Treatment of aneurysmal subarachnoid hemorrhages

How is hemodynamics usually controlled? (multiple answers possible)

- About arterial pressure curve measurement
- Via extended hemodynamic monitoring e.g. PiCCO catheter
- Regular POCUS ultrasound examination (cardiac echocardiography, vena cava ultrasound)
- Other

11. Treatment of aneurysmal subarachnoid hemorrhages

At what hemoglobin level does red blood cell substitution take place in patients you treat with aneurysmal subarachnoid hemorrhage?

- Hb value < 7 mg/dl

- Hb value < 8 mg/dl
- Hb value < 9 mg/dl
- Hb value < 10 mg/dl
- There is no concrete value that triggers transfusion.

11. Treatment of aneurysmal subarachnoid hemorrhages

If a delayed ischemic deficit is detected, at what hemoglobin level does red blood cell substitution take place in patients you treat with aneurysmal subarachnoid hemorrhage?

- Hb value < 7 mg/dl
- Hb value < 8 mg/dl
- Hb value < 9 mg/dl
- Hb value < 10 mg/dl
- There is no concrete value that triggers transfusion.

11. Treatment of aneurysmal subarachnoid hemorrhages

How is core body temperature measured in your intensive care unit? (Multiple answers possible)

- tympanum
- Intraesophageal
- Intravesical
- Continuously
- Discontinuous
- Several locations
- Other

11. Treatment of aneurysmal subarachnoid hemorrhages

At what body temperature are temperature-reducing measures initiated?

- ≥38.5◦C
- ≥38◦C
- ≥37.5◦C
- No routine temperature reduction
- Other

11. Treatment of aneurysmal subarachnoid hemorrhages

Other

Answer

12. Treatment of aneurysmal subarachnoid hemorrhages

Is prophylactic controlled normothermia used in your intensive care unit?

- Yes
- No

12. Treatment of aneurysmal subarachnoid hemorrhages

What measures are taken to control the temperature? (multiple answers possible)

- Cold infusions
- calf wrap/peppermint wash
- Medicinal fever reduction with NSAIDs, paracetamol, metamizole
- cooling blanket/cooling pads
- Mechanical techniques (feedback-controlled devices such as adhesive pads,
- catheter cooling)
- Other

12. Treatment of aneurysmal subarachnoid hemorrhages

Other

Answer

12. Treatment of aneurysmal subarachnoid hemorrhages

If delayed ischemic deficit occurs, are platelet function inhibitors used for prophylaxis?

- Yes
- No

12. Treatment of aneurysmal subarachnoid hemorrhages

From which day are patients with aneurysmal subarachnoid hemorrhage fed?

- From day 1
- From day 2
- From day 3
- Other

12. Treatment of aneurysmal subarachnoid hemorrhages

Other

Answer

12. Treatment of aneurysmal subarachnoid hemorrhages

Which food is preferred in terms of calories for patients with aneurysmal subarachnoid hemorrhage?

- Hypocaloric food
- Normocaloric diet
- Hypercaloric food

12. Treatment of aneurysmal subarachnoid hemorrhages

How early are patients with aneurysmal subarachnoid hemorrhage mobilized?

- Immediately after aneurysm treatment, if there are no contraindications
- Within the first 24-72 hours
- > 72 hours
- Other

12. Treatment of aneurysmal subarachnoid hemorrhages

Other

Answer

12. Treatment of aneurysmal subarachnoid hemorrhages

When is a tracheostomy performed in severely affected patients?

- After less than 7 days after SAB
- > 10 – 14 days after SAB
- Individually according to the team's decision

12. Treatment of aneurysmal subarachnoid hemorrhages

If the initial DSA does not detect an aneurysm as the cause of bleeding, when is a follow-up DSA performed?

A In cases of aneurysmal-seeming subarachnoid hemorrhage

- follow-up examination after 1 week

- follow-up examination after 2 - 4 weeks
- follow-up examination after 6 weeks
- No follow-up examination after high-quality initial examination
- Other

B In case of purely prepontine/perimesencephalic bleeding

- follow-up examination after 1 week
- follow-up examination after 2 - 4 weeks
- follow-up examination after 6 weeks
- No follow-up examination after high-quality initial examination
- Other

12. Treatment of aneurysmal subarachnoid hemorrhages

If you selected "Other", please explain here:

Answer

12. Treatment of aneurysmal subarachnoid hemorrhages

Treatment of aneurysmal subarachnoid hemorrhages

- **1:1**
- 1:1
- 1:2
-
- **2:1**
- 1:1
- 1:2
